# The psychometric properties of the Chinese version of the Parkinson Anxiety Scale (C-PAS) among Parkinson’s disease

**DOI:** 10.1186/s12883-023-03095-9

**Published:** 2023-02-04

**Authors:** Yuanyuan Jiang, Yelin Jiang, Tiantian Peng, Mengyue Wang, Manman Li, Min Zhang, Li Li, Qi Zhang

**Affiliations:** 1 Department of Neurology, Nanjing Drum Tower Hospital, The Affiliated Hospital of Nanjing University Medical School, No.321 Zhongshan Road, Gulou District, 210008 Nanjing, China; 2 School of Nursing, Peking University, Beijing, China

**Keywords:** Parkinson disease, Anxiety, Psychometric properties, Validity

## Abstract

**Background:**

Anxiety disorder is a common non-motor symptom among individuals with Parkinson’s disease (PD). At present, there are no specific tools in China for assessing the anxiety level of patients with PD. This study aimed to test the reliability and validity of the Chinese version of the Parkinson Anxiety Scale (C-PAS) in Chinese patients with PD.

**Methods:**

A total of 158 patients with PD at one hospital in Nanjing were recruited through convenience sampling. The C-PAS was translated into Chinese using a classic ‘forward-backward’ translation method. Reliability tests included internal consistency and test-reliability. And in addition to content, structure and criterion-related validity were performed for the validity tests. Criterion-related validity was evaluated with the Hospital Anxiety and Depression Scale-Anxiety Subscale (HADS-A).

**Results:**

Results confirmed the three-factor structure of the original C-PAS with 12 items, including persistent anxiety (5 items), episodic anxiety (4 items) and avoidance behavior (3 items). Significant and positive correlations were obtained between C-PAS and HADS-A (*r* = 0.82, *P*<0.01). The Cronbach’s α and test-retest reliability of the total scale were 0.89 and 0.84, respectively.

**Conclusion:**

The C-PAS has demonstrated good psychometric properties. Therefore, it can be employed in patients with PD to evaluate the condition of anxiety.

## Background

Anxiety disorder, which may present as panic attacks, phobias, or generalized anxiety disorder [1], is a common non-motor symptom among individuals with Parkinson’s disease (PD) [2]. Anxiety is characterized by the feeling of worry, lack of concentration, muscle tension, headache, and insomnia [3], and can be episodic or nonepisodic, often worsening during off periods or continuous [4]. Approximately 31% of PD patients experienced an anxiety disorder [5], higher than patients with other diseases. Anxiety is a known contributor to accelerated disability and functional morbidity and associates with poor prognosis, reduced quality of life in patients with PD, increasing caregiver burden and health-related costs [6]. Currently, the prevalence of anxiety is high in Chinese patients with PD, although it is often neglected and undermanaged [7, 8]. However, there is no standard instrument for measuring anxiety level among patients with PD in China, which may lead to anxiety remaining unmeasured and undiagnosed in PD patients. Hence, early detection, accurate assessment and adequate treatment may substantially alleviate anxiety level in patients with PD [4].

Anxiety is a subjective cognitive and emotional experience [9]. A valid and reliable scale has the advantage in helping PD patients find their “hidden” anxiety problems and freeing up clinicians’ time for more in-depth diagnostic interviews. Worldwide, a large number of general anxiety instruments have been validated in patients with PD, such as the Beck Anxiety Inventory, the Hospital Anxiety and Depression Scale, the Zung Self-rating Anxiety Scale, Anxiety Status Inventory, the Spielberger State-trait Anxiety Inventory, and the Hamilton Anxiety Rating Scale [1, 8, 10, 11]. Although these instruments have been psychometrically evaluated and frequently used for assessing anxiety levels in clinical and research settings, generic anxiety instruments do not allow assessing specific aspects of anxiety in samples of individuals with PD [12]. Furthermore, a previous study also showed that some physical symptoms of PD might overlap with symptoms of anxiety disorders and further decrease their validity [1]. Hence, instruments that are specific for PD patients should be used to screen PD-related anxiety.

To overcome the limitations of existing instruments, the Parkinson Anxiety Scale (PAS) was specifically developed by Leentjens et al. [13] to assess the anxiety in PD patients. This novel PAS has demonstrated psychometric superiority over general anxiety rating scales in the Netherlands. Furthermore, this 12-item PAS was brief and easy to administer in clinical practice and has been translated into Italian with acceptable reliability and validity in PD patients [14]. However, the Chinese version of the PAS (C-PAS) has yet to be validated for use with Chinese individuals with PD. The psychometric properties of the C-PAS are therefore urgently required to inform its use in future research and clinical practice.

The aim of our study was to assess the validity and reliability of the PAS among patients with PD in Chinese mainland. We began by conducting exploratory factor analysis (EFA) to explore the factor structure of C-PAS in Chinese individuals with PD. We hypothesized that the C-PAS factors would demonstrate adequate internal consistency and stability, would differentiate between PD patients with and without an anxiety, and would demonstrate adequate content validity evaluated by a six-number expert panel and criterion-related validity by correlating with measures of Hospital Anxiety and Depression Scale-Anxiety Subscale (HADS-A).

## Methods

### Design and participants

A cross-sectional study was carried out to evaluate the reliability and validity of the C-PAS. Using a convenience-sampling strategy, we recruited patients with PD from neurology clinic in one hospital in Nanjing, from September 2020 to July 2021. As a rule of thumb, at least 10 respondents were involved in each item of the exploratory factor analysis [15]. A sample size of a minimum of 144 was figured out/define because the C-PAS included 12 items. The eligible criteria that every participant must meet are as follows: 1) aged 18 to 80 years old; 2) a diagnosis of PD according to the criteria of Movement Disorder of Society [16]; 3) having the ability to read and write mandarin Chinese; 4) agreeing to participate and sign informed written consent. Patients who met the inclusion criteria read and answered the questions independently. If the patients did not understand, the researchers provided explanations to help complete questionnaires.

According to the ethical rules of the Helsinki Declaration, our study was approved by the Ethics Committee of Hospital. Written informed consent was acquired from all participants, and the information was ensured to be confidential.

### Measurements

All data were collected by a face-to-face interview during a one-time interview in outpatient clinics.

#### Socio-demographic and clinical characteristics

The socio-demographic information included age, gender, educational degree, and so on. Clinical characteristics were evaluated by asking the duration of PD and other chronic disease.

#### C-PAS

The PAS was designed by Dr. Leentjens to measure the level of anxiety, especially in PD patients, and it can also be used in individuals with other neurological disorders [13]. The instrument covered three subscales: persisting anxiety subscale to evaluate generalized anxiety disorders (five items), episodic anxiety subscale to evaluate panic disorders (four items), and avoidance behaviour subscale to evaluate combined symptoms of social phobia and agoraphobia (three items). Each item is rated on a 5-point Likert-type scale (0 = ‘not or never’ to 4 = ‘severe or almost always’). The higher scores indicate more serious anxiety. In the study conducted with 362 patients with PD, Cronbach’s α (0.89) was acceptable.

#### The Chinese version of HADS-A

The HADS-A was developed by Zigmond and Snaith and used to assess symptoms of anxiety in general patients. The anxiety subscale has seven items, each being a 4-point Likert scale ranging from 0 to 3 [17]. The reliability and validity of this instrument in the Chinese version have been assessed, showing good Cronbach’s α of 0.88 [18]. Therefore, the Chinese version of the HADS-A had good psychometric properties. The HADS-A was used to evaluate criterion-related validity of C-PAS.

### Translation and adaptation of the PAS

We obtained permission to translate and apply this instrument from Dr. Leentjens. After that, we used a classic ‘forward-backward’ translation method to translate the PAS into mandarin Chinese [19, 20], involving translation, back-translation, and cultural adaptation. First, the initial English version of PAS was independently translated into mandarin Chinese by two researchers, a nurse with a doctorate and a head nurse with overseas study experience in the neurology department, and the two researchers reached an agreement on the initial draft of the C-PAS. Then, two bilingual individuals who were both neurological physicians with experience of studying abroad translated the C-PAS back into English. Moreover, a six-member expert panel including two neurological physicians, two neurological head nurses, a professor of chronic disease management and a professor of psychological nursing analyzed differences between translation and the English version, and then modified the text further to keep appropriateness for both cultures as well as feasibility of the C-PAS. Finally, pilot testing (*n* = 20) was performed to examine the language of the C-PAS and PD patients’ understanding of the C-PAS. We established the final C-PAS, and all items were easy to understand for PD patients.

### Statistical analysis

Statistical analysis of all data was conducted by SPSS 26.0 software. Descriptive analyses were used with data that involved socio-demographic and clinical participants’ characteristics. The statistical significance level was set at 0.05.

Percentage of missing or invalid items (<5% is acceptable) and extent of ceiling and floor effects of total and subscales (<15% were defined as optimal) were analyzed to evaluate the quality of data. We adopted internal consistency and test-reliability to assess the reliability of the C-PAS. Internal consistency was examined by Cronbach’s α of which 0.70 or above was considered acceptable, and corrected item-total correlation of which 0.40 or above was considered acceptable [13].

In order to guarantee the stability of the C-PAS, we conducted the test-retest survey in 20 persons with PD after 2 weeks of the initial test. The stability of the C-PAS was assessed by test-retest reliability applying to compute the intra-class correlation coefficient (ICC) for the total scale and three subscales.

ICC is a more desirable measure of reliability reflecting both degree of correlation and agreement between measurements [21]. We used two-way mixed-effects model to access the test-retest reliability, as our results only represent the reliability of the specific raters involved in the reliability experiment but no other raters. ICC values higher or equals to 0.75 were considered good [21].

The evaluation of validity for the C-PAS consisted of content validity, criterion-related validity, and construct validity. Content validity refers to the relevant degree of the actual measured content and the target measured content in an instrument, which is evaluated by calculating the content validity index (CVI) [22, 23]. In this study, the six-number expert panel measured each item to verify relevance, clarity, and simplicity according to a 4-point Likert scale ranging from 1 (irrelevant) to 4 (strongly relevant). Both scale-level content validity index (S-CVI) and item-level content validity index (I-CVI) were calculated to assess the content validity of the C-PAS. I-CVI is regarded as the proportion of experts that scored ‘3’ or ‘4’ for each item, and S-CVI is regarded as the proportion of items rated ‘3’ or ‘4’ by all experts [24]. S-CVI > 0.80 and I-CVI ≥ 0.78 indicated that content validity in this study was satisfactory in the instrument [25]. Criterion-related validity is defined as being highly correlated with the structure measured by instruments [26]. We hypothesized that there was a significant correlation between C-PAS and the HADS-A in patients with PD. Spearman rank correlation coefficients were used to assess criterion-related validity between the C-PAS and the HADS-A, with correlation coefficients of > 0.60 was considered to be high. Construct validity was evaluated by EFA. EFA employed Bartlett’s Test of Sphericity, and the Keiser-Meyer-Olkin index (KMO) was performed to examine the compatibility of data for factor analysis. A KMO index of > 0.70 is suitable to evaluate EFA. We used principal component analysis with Varimax rotation to explore the structure of the C-PAS, in which the number of factors was concluded by a scree plot and eigenvalues > 1.

## Results

### Characteristics of the participants

Of the 158 participants recruited in our study, 150 participants completed the questionnaires (response rate was 95%). The socio-demographic and clinical characteristics of the participants are described in Table 1.

**Table 1:** Demographic and Disease Characteristics of Participants

Variables	N (%) / Mean ± SD
**Sex**	
Male	91 (60.7%)
Female	59 (39.3%)
**Age**	65.21 ± 8.59
**Educational degree**	
Middle school or under	83 (55.3%)
High school or technical secondary school	39 (26%)
College or above	28 (18.7%)
**Marital status**	
Married	139 (92.6%)
Others (divorced/ bereaved)	11 (7.4%)
**Living arrangements**	
Living with family member	142 (94.6%)
Others (alone/ with nursing workers)	8 (5.4%)
**Employment**	
Working	26 (17.3%)
Retired	95 (63.3%)
Others	29 (19.4%)
**Household income (Yuan/month)**	
< 5000	60 (40%)
5000–10,000	59 (39.3%)
> 10,000	31 (20.7%)
**Medical insurance**	
Yes	133 (88.6%)
No	17 (11.4%)
**Other chronic diseases**	
Yes	130 (86.7%)
No	20 (13.3%)
**Duration of PD (Years)**	
1–4	125 (83.3%)
5–9	21 (14%)
≥ 10	4 (2.7%)

### Quality of data

In terms of quality of data, there was only 5% missing items in our study. There were no floor (0%) and ceiling effects (3%) for C-PAS. No ceiling effect was found for all subscales, whereas floor effect was found for the episodic (16%) and avoidant (29%) subscales.

### Reliability

Cronbach’s α value was 0.89 of the total scale, 0.90 for the persisting subscale, 0.84 for the episodic subscale, and 0.73 for the avoidance subscale. The inter-item correlations of all items varied from 0.16 to 0.73, and item-total correlations of all items varied from 0.29 to 0.78, indicating a high correlation. The C-PAS had good test-retest reliability with a total ICC of 0.84 at first and 2 weeks later evaluations, as is showed in Table 2.

**Table 2:** Test-retest reliability of total scale and subscales

Items	ICC	95% CI	*P*
Persisting anxiety subscale	0.88	0.63; 0.96	<0.01
Episodic anxiety subscale	0.68	0.35; 0.86	<0.01
Avoidance behaviour subscale	0.73	0.43; 0.88	<0.01
Total scale	0.84	0.64; 0.94	<0.01

### Validity

The C-PAS had acceptable content validity with the S-CVI value of 0.97 and the I-CVI statistics varying from 0.83 to 1.00. The significant and positive correlation between the C-PAS and the HADS-A demonstrated that the criterion-related validity was satisfactory (*r* = 0.82, *P*<0.01). Both significant results of Bartlett’s sphericity test (*χ2* = 1005.540, df = 66, *P*<0.01) and the KMO index of 0.85 suggested sampling adequacy in EFA. The three factors constituted 69.9% of the total variance. The factor loading of item 6 had a correlation with both the persisting and the episodic factors. Finally, we determined to retain this item and accept the contribution after discussion with experts (Table 3).

**Table 3:** Factor Loading in Exploratory Factor Analysis and Scale Reliability

Item	Median	Inter-quartile Range	Corrected Item-total Correlation	Cronbach’s Alpha (if item deleted)	Factor Loading		
					1	2	3
1	1.00	0.00–2.00	0.78	0.87	**0.74**	0.29	0.37
2	1.00	0.00–2.00	0.75	0.87	**0.71**	0.25	0.39
3	1.00	0.00–2.00	0.70	0.87	**0.69**	0.20	0.38
4	1.00	0.00–1.00	0.65	0.88	**0.84**	0.17	0.11
5	1.00	0.00–1.25	0.66	0.88	**0.79**	0.22	0.14
6	0.00	0.00–1.00	0.58	0.88	**0.42**	**0.76**	−0.10
7	1.00	0.00–2.00	0.56	0.88	0.16	**0.81**	0.19
8	1.00	0.00–2.00	0.48	0.89	0.06	**0.84**	0.14
9	1.00	0.00–2.00	0.57	0.88	0.33	**0.69**	0.07
10	1.00	0.00–1.25	0.54	0.88	0.17	0.14	**0.91**
11	1.00	0.00–1.00	0.59	0.88	0.21	0.19	**0.86**
12	0.00	0.00–1.00	0.29	0.90	0.21	−0.05	**0.49**

## Discussion

Anxiety is a disabling disorder, which causes social stigmatization, professional exclusion, poverty and represents a growing economic burden for societies [10]. The psychometric property of the C-PAS was promising, with sufficient validity and satisfactory reliability in PD patients. The three-factor structure of C-PAS was found to correspond with the original version. All subscales had good internal consistency and stability in the samples with PD, as predicted. Similarly, the total C-PAS had good internal consistency and stability, as predicted. With regard to content validity in the PD, as predicted, the content and criterion validity were both acceptable. The popularization and standardized application of C-PAS are anticipated to be valuable in homogenization comparison between different ethnic groups. Additionally, the C-PAS was undertaken strictly in accordance with established guidelines and the latest method of adaptation [27]. All questionnaire items were accurate, comprehensible, and easy to answer for participants.

In this study, the C-PAS showed good content validity, with the I-CVI>0.78 and the S-CVI = 0.97. As in our previous hypothesis, criterion validity was acceptable due to a strong and positive correlation between the C-PAS and the HADS-A. The total variance of the C-PAS corresponds to the standard required for the structural validity of the C-PAS. However, the item “In the past four weeks, did you experience episodes of panic or intense fear” concurrently had high factor loadings on factor 1 (persistent anxiety) and factor 2 (episodic anxiety). According to the results of concept analysis and expert consultation, we finally retained and categorized it into factor 2 (episodic anxiety). Hence, there was also a three-factor structure in C-PAS: namely persistent anxiety (5 items), episodic anxiety (4 items) and avoidance behavior (3 items). The final three-factor structure was consistent with the original version.

Good reliability was found in our study, as indicated by the excellent internal consistency and good test-retest reliability. Results from our study supported the acceptable internal consistency. The Cronbach’s α of the C-PAS (0.89) were consistent with the original version (0.87 for patient-rated version) [13]. This result was also consistent with previous reports of Italian observer-rated version of the PAS, with good internal consistency of total scale (0.90) and all subscales (from 0.75 to 0.84) [14]. In addition, the test-retest reliability of C-PAS after a 2-week period was preferable (ICC = 0.84) in our study, which was also consistent with the original self-rated version (ICC = 0.89) [13].

Several limitations regarding the findings of this work should be considered. Firstly, the participants in this study were recruited from only one hospital in China, which might influence the generalization of our results. Secondly, data of disease stage that evaluated by clinical assessment scales, such as the Unified Parkinson’s Disease Rating Scale and the Hoehn & Yahr staging system were lacking, which limited further analyses of the applicability of C-PAS in patients with different disease severity. Thirdly, differences in demographic characteristics, such as age and duration of disease among patients may affect the evaluation of measurement properties for C-PAS. Lastly, we did not employ the clinical diagnostic criteria of anxiety for identifying patients with anxiety disorder and providing diagnostic cut-off scores for C-PAS. In this regard, future studies should use a standard gold diagnosis to further verify the value of C-PAS in diagnosis of anxiety disorder among patients with PD for further diagnostic and therapeutic work-up in Chinese PD patients.

Despite the limitations, this investigation adds evidence for the reliability and validity of C-PAS and indicates the C-PAS is a psychometrically sound measure to assess anxiety in individuals with PD. To our knowledge, this is the first study that analyses the psychometric properties of such a brief anxiety instrument designed specifically for PD patients in China. The study fulfilled an important gap in the current pool of developmentally sensitive anxiety assessment instruments in China and showed potential practical implications for screening practice in PD patients. Given its brief format, the C-PAS may be used in multiple circumstances, including assessing PD, guiding educational efforts and exploring the relationships between anxiety and other variables in PD patients.

## Conclusion

In summary, the C-PAS exhibited good reliability and validity. This standardized instrument could be of use to service providers for the screen of PD patients and to provide timely individual interventions to improve their emotional state in China. Nevertheless, further research also needs to be conducted on a larger scale.

## Data Availability

The datasets generated and analysed in the current study are available from the corresponding author on reasonable request.

## Abbreviations

C-PAS: Chinese version of the Parkinson Anxiety Scale
PD: Parkinson’s disease
HADS-A: Hospital Anxiety and Depression Scale-Anxiety Subscale
PAS: Parkinson Anxiety Scale
EFA: exploratory factor analysis
ICC: intra-class correlation coefficient
CVI: content validity index
S-CVI: scale-level content validity index
I-CVI: item-level content validity index
KMO: Keiser-Meyer-Olkin index
